# Exploring Quality of Life and Mortality in Pertrochanteric Fragility Hip Fractures in Northern Greece: A Single Tertiary Center Study

**DOI:** 10.3390/jcm13092478

**Published:** 2024-04-24

**Authors:** Panagiotis Konstantinou, Lazaros Kostretzis, Georgios Fragkiadakis, Panagiota Touchtidou, Argyrios Mavrovouniotis, Vasileios Davitis, Athina Zacharoula Ditsiou, Ioannis Gigis, Anastasios P. Nikolaides, Dimitris Niakas, Pericles Papadopoulos, Konstantinos Ditsios

**Affiliations:** 1 2nd Orthopaedic Department, Aristotle University of Thessaloniki, “G. Gennimatas” Hospital, Eth. Aminis 41, 546 35 Thessaloniki, Greece; lazaros.kostretzis@gmail.com (L.K.); touhtidou.p@hotmail.com (P.T.); armavrovo@gmail.com (A.M.); vasilisdavitis@hotmail.com (V.D.); jgigis71@gmail.com (I.G.); perpap@otenet.gr (P.P.); ditsiosk@otenet.gr (K.D.); 2 University Hospitals Birmingham NHS Foundation Trust, Birmingham B7 5TE, UK; anastasios.nikolaidis@uhb.nhs.uk; 3 Healthcare Management, School of Social Science, Hellenic Open University, 263 35 Patra, Greece; fragkiadakis.georgios@ac.eap.gr (G.F.); dniakas@med.uoa.gr (D.N.); 4 School of Medicine, Faculty of Health Sciences, Aristotle University of Thessaloniki, 541 24 Thessaloniki, Greece; ditsiouathina@gmail.com

**Keywords:** health related quality of life, fragility fractures, hip fracture, mortality, SF-12, EQ-5D

## Abstract

**Background:** Fragility-related pertrochanteric fractures have become a significant public health concern, with a rising incidence attributed to the expanding elderly demographic. Assessing patient-reported health-related quality of life (HRQoL), mortality, and factors correlated with them serves as a crucial metric in evaluating the effectiveness of hip fracture surgery. **Methods:** In a single-center retrospective study, 259 patients underwent surgical treatment with a cephalomedullary nail, with a mean follow-up of 21.7 months. Health-related quality of life (HRQoL) was assessed using SF-12 (12-item Short Form) and EQ-5D (EuroQoL-5 Dimensions) questionnaires. Mobility status was measured by the Crude Mobility Index (CMI). Surveys were administered during hospitalization and six months postoperatively. Statistical analysis involved descriptive statistics, non-parametric controls (Kendall, Mann-Whitney, and Wilcoxon), and Spearman correlation and logistic regression analysis, which were conducted using IBM SPSS version 28. **Results:** A statistically significant decrease was observed in the mean EQ-5D and SF-12 scores at 6 months post-op compared to the pre-fracture status. The ASA (American Society of Anaesthesiologists) score showed a significant correlation with the decrease in HRQoL measured by the SF-12 questionnaire. The 30-day post-operative mortality rate was 9.3%, increasing to 32.4% at 1 year. Notably, the 30-day mortality significantly rose during the pandemic era (5.0% vs. 12.0%; *p* = 0.003). **Conclusions:** Pertrochanteric hip fractures cause a lasting decline in quality of life. Annual mortality is high, and further investigations are needed to formulate policies that prevent hip fractures and reduce mortality rates.

## 1. Introduction

The incidence of intertrochanteric fractures in the year 2019 was 304 cases per 100,000 inhabitants in the US population [1]. With the increasing life expectancy of the general population, fractures in the elderly are also anticipated to rise, with several prognoses estimating their number to reach 4.5 million by 2050 [2]. These fractures involve extracapsular injuries occurring between the greater and lesser trochanter. These fractures are typically managed through internal fixation with intramedullary fixation devices or a dynamic hip screw (DHS) to facilitate early ambulation, aiming to reduce the risk of complications and mortality [3]. The associated costs of hospitalization and one-year rehabilitation are enormous for society, accounting for approximately 43,000 USD per patient [4].

The 1-year mortality rate for intertrochanteric fractures ranges from 34% to 23% in the literature [5]. Managing this type of fracture poses a significant challenge due to the limited number of patients returning to their usual daily routines and performing typical tasks post-surgery [6]. Approximately half of patients require assistance with their daily activities, and around 25% necessitate long-term care [7]. While most studies focus on fracture union, surgical complications, and revisions, little attention is shown to quality of life metrics, which actually reflect the decrease in patients’ well-being and its possible reversibility [8,9,10,11,12].

Since valid nationwide registry data for intertrochanteric fractures and quality of life studies are lacking in Greece, our purpose was to retrospectively record all patients with intertrochanteric fractures from 2019 to 2021 to evaluate their quality of life postoperatively. Secondly, we aimed to analyze the 30-day and 1-year mortality rates, transfusion rate, and mobility status of our patients. Additionally, given that our study coincided with the COVID-19 pandemic, we decided to compare differences in mortality rates between the pre-COVID era and the pandemic period. We hypothesized that intertrochanteric fractures would have a negative impact on health-related quality of life metrics and that mortality would increase during the COVID-19 era.

## 2. Materials and Methods

### 2.1. Study Design and Patients

This is a single-center, retrospective observational study focusing on patients who sustained a trochanteric hip fracture between January 2019 and November 2021. Our hospital, located in a city with a population of 1.5 million, is on 24-h duty every 4 days, which means 7 to 8 times per month. Additionally, it serves as a reference center for more complicated cases, handling fractures of increased complexity or patients with higher frailty levels within the region of Northern Greece (central Macedonia). The inclusion criteria were age above 65, acute trochanteric hip fracture (S72.1), based on the International Classification of Diseases (ICD-10), and surgical treatment with cephalomedulary nails. Patients with any form of cognitive impairment or lingual barrier were excluded, as these conditions may impede their understanding of the questionnaires. The procedures were compliant with the Declaration of Helsinki. All patients signed an informed consent form, and the study was approved by the hospital’s Clinical Research Committee (2^η^/24-3-2023 No 21).

**Chart 1:**
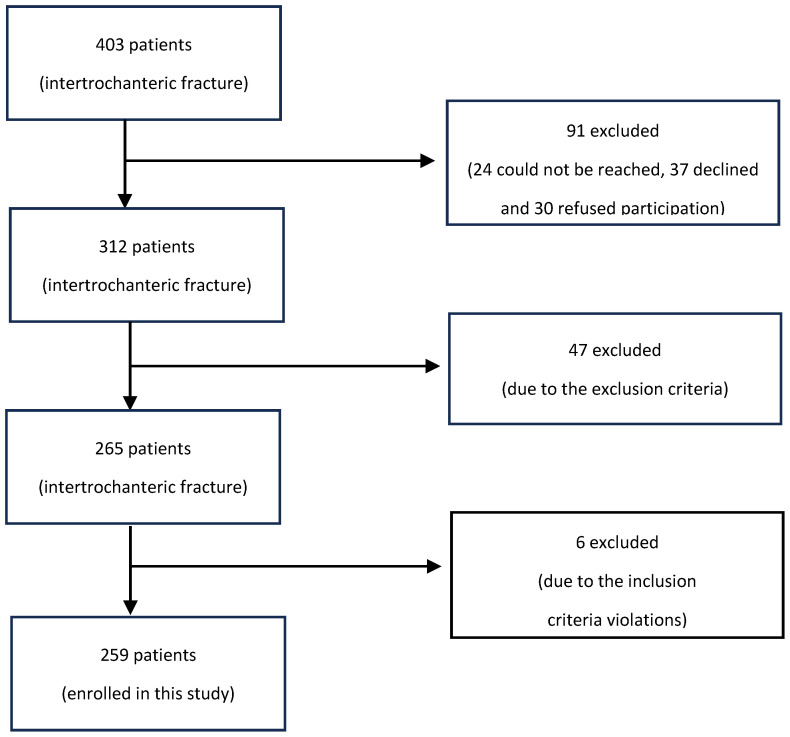
Flowchart of patients enrolled the study.

A total of 403 patients were admitted for intertrochanteric hip fractures during the study period. Nighty-one patients could not be reached, declined, or refused to participate, and an additional 47 were excluded due to the exclusion criteria. The remaining 265 patients were enrolled in our study. After data verification, six more patients were excluded due to inclusion criteria violations. The final study population consisted of 259 patients (Chart 1). The mean follow-up was 21.7 (0–49) months. The COVID era was defined as the period from March 2020 until the end of the study, considering that the first COVID-19 case in Greece was reported on 26 February 2020, and the government announced a lockdown in March 2020.

### 2.2. Data Collection

HRQoL was evaluated using two questionnaires, the SF-12 [13] and the EuroQol-5D [14]. Both questionnaires have been translated into the Greek language, and their validity has already been tested in the Greek population [15,16]. The SF-12 is a brief health survey evaluating eight health domains: physical functioning, role-physical, bodily pain, general health, vitality, social functioning, role-emotional, and mental health. Scores from each health domain contribute to the overall Physical Component Summary (PCS) and Mental Component Summary (MCS) scores. Scores vary between 0 and 100, with elevated scores signifying superior physical and mental health functioning. The EuroQol-5D questionnaire is a descriptive system comprised of five domains (mobility, self-care, regular activities, pain/discomfort, and anxiety/depression), each one with five levels of severity (denoted as 1–5, where 1 indicates no problem and gradually worsen till 5, which indicates extreme problems). Additionally, it incorporates a visual analog scale (EQ-5D VAS), represented by a 20 cm vertical scale with the extreme expression of self-perceived health at either end. This scale ranges from 0 (worst health) to 100 (best health). Both SF-12 and EQ-5D questionnaires have been utilized in several studies assessing the quality of life in patients with hip fractures [11,17,18,19,20]. For the physical fitness classification, the American Society of Anesthesiologists (ASA) scale [21] was utilized, and for the assessment of patients’ comorbidities, the Charlson Comorbidity Index (CCI) was calculated [22]. This Index is a predictive model in nature, assigning numerical values to various chronic conditions and deriving the overall score for each individual patient by summing up the partial values.

To assess the mobility status of patients, we introduced and used a five-grade scale, categorizing them based on their mobility status, which was one week prior to the fracture and six months post-op. We characterize mobility as 1/5 if the patient was bedridden, 2/5 if they were able to walk small distance with the help of a person/walker/stick (inside house), 3/5 for those who walked with walker/rollator or stick, 4/5 for patients walking independently indoors and outdoors but not for long distances and 5/5 for those who had no limitation of mobility. We named this index as Crude Mobility Index (CMI).

The two HRQoL surveys were administered and completed during patients’ hospitalization, assessing their pre-fracture status one week prior to the fracture. The follow-up questionnaire was conducted six months later via a phone call by authors A.M., V.D., A.D. The mobility status was assessed the same way, at the admission and six months later. The remaining data needed for our study was extracted by reviewing the patients’ hospital medical records.

### 2.3. Surgical Technique

All fractures were treated in the same hospital by our department’s surgeons, using the Dyna Locking Trochanterix (DLT) short nail. The reduction of these fractures was conducted in a closed manner or minimally invasive on the traction table, and no fracture was reduced in an open manner. We used a Proximal screw and compressed the fractures with dynamic locking of the proximal screw. Distally we lock the nail with one static screw. The cut-off limit for blood transfusion was hemoglobin levels below 8.5 g/dL, except for patients with renal and cardiologic issues, for whom this threshold was set at 10 g/dL.

### 2.4. Statistical Analysis

The incidence of pertrochanteric fracture in our population was found to be 79.3 cases per 100,000 population. There is no registry for these types of injuries in Greece, and we calculated the incidence based on our annual number of operations and the prefecture’s population, according to the latest population count in 2021 [23]. It is important to note that our department operates on a 24-h duty schedule every four days. For the descriptive analysis, we computed percentages for categorical variables and determined the mean with range for continuous variables such as age, transfusion units, ASA, and the Charlson Comorbidity Index. Normal distribution consistency was assessed using the Kolmogorov-Smirnov test, and depending on whether variables followed this distribution or not, the Student’s *t*-test or Mann-Whitney U-test was applied for quantitative variables, while the Chi-square test or Fisher’s exact test was utilized for categorical variables as appropriate. The relationship between quantitative variables was examined using Spearman’s Rho correlation coefficient. Paired groups (pre-fracture status/six-month comparison) were analyzed using the Wilcoxon signed-rank test for continuous variables (SF-12, EQ-5D), and the Pearson chi-squared test was employed for comparing categorical variables. All the above statistical analyses were performed with IBM SPSS package version 28.

## 3. Results

### 3.1. Baseline Characteristics

The clinical and socio-demographic characteristics of the patients are presented in Table 1a. The mean age of the admitted patients was 83.6 years, and women were, on average, 2.3 years older than men. The gender ratio was almost 4:1, women to men. The mean ASA score was 3 (2–4), and the mean Charlson Comorbidity Index was 5.7 (2–13). The vast majority of patients (83.8%) were non-smokers and lived in their homes (94.6%) before the hospital admission. The percentage of those who were undertaking osteoprotective treatment at the time the fracture occurred was significantly low (3.9%). The mean time elapsed between admission and operation was 2.7 days (0–11). In addition, the mean total length of stay was 7.5 days (1–23). A percentage of 69.1% of all patients chose to continue their rehabilitation in an organized rehabilitation center after their hospital discharge. Moreover, a significant increase was observed in the percentage of institutionalized patients from the pre-fracture status (5.4%) to 30 days post-op (69.1%), *p* < 0.001. The corresponding patient characteristics, based on pre-and post-COVID-19 periods, are presented in Table 1b.

### 3.2. Health-Related Quality of Life

The total number of patients evaluated for quality of life is 190 (73.4%), as 69 (26.6%) patients died within six months after the surgical treatment without completing the relevant questionnaires. Concerning QoL (quality of life), the mean score for the EQ-5D index and EQ-5D VAS showed a significant decrease six months after the operation. The scores dropped from 0.74 (0.48–0.87), representing the pre-fracture status, to 0.39 (0.17–0.72) (*p* < 0.001) for EQ-5D index and from 69.7 (46–89) to 42.4 (28–61) (*p* < 0.001) for EQ-5D VAS, respectively. The assessment of HRQoL using the SF-12 questionnaire led to the same conclusion: a significant decrease in quality of life for patients six months post-op. The physical component dropped from 34.2 (27.3–54.5) to 27.6 (17.1–49.7) (*p* < 0.001), and the mental component fell from 54.49 (39.5–71.7) to 38.6 (27.2–67.3) (*p* < 0.001) (Table 2a).

In univariate analysis, only ASA score was found to be significantly correlated with Quality of Life measured by SF-12 (r = 0.232, *p* = 0.018). Age and Charlson Comorbidity did not significantly affect the quality of life six months after the operation (see Table 3). The analysis showed that there is no significant difference in the quality of life based on the EQ-5D index, whether patients received rehabilitation at home (U = 1009.5, *p* = 0.543) or received services in a rehabilitation center (U = 864.0, *p* = 0.224). The same conclusions for QoL were applied by evaluation with the SF-12 questionnaire at home (U = 1091.0, *p* = 0.977) and in a rehabilitation center (U = 944.5, *p* = 0.540) (Table 2b).

### 3.3. Mortality

A total of 259 patients with an intertrochanteric fracture were followed up for an average time of 21.7 (0–49) months. The 30-day mortality was 9.3%, with rates of 7.5% for women and 17% for men. The 30-day mortality was 5% in the non-COVID era and rose to 12% during a pandemic. This increase was significant (x^2^ = 8.72, *p* = 0.003). The annual mortality for this type of fracture was 32.4%, with rates of 33.5% for women and 27.7% for men (*p* = 0.21) (Table 3).

Statistical analyses revealed a significant correlation between the ASA score and 30-day mortality (x^2^ = 8.546, *p* < 0.01), and all the other tested parameters (CCI, gender, transfusion) showed no significant correlation with mortality at 30 days and one year follow up (Table 4). Finally, lower levels of hemoglobin at admission or on discharge and age above 85 were found to be significantly higher in the mortality group than in the survival group at 12-month follow-up. The time from injury to operation has no significant difference between the mortality and survival group (Table 5).

### 3.4. Transfusion

Several characteristics were studied between the group of patients who received transfusion and the group without transfusion. The transfusion rate was found to be 66.4%, as 172 out of 259 patients received at least 1 unit of blood. In our study, blood transfusion was found to be related to age and hemoglobin, as a significant difference was noted in age, admission hemoglobin, and discharge hemoglobin between the transfused and non-transfused groups. The time elapsed from injury to surgical treatment did not affect the possibility of transfusion, as the difference between the two groups was not significant (Table 6).

### 3.5. Mobility Six Months Post-Op

Regarding the mobility of the patients, a significant decrease was noted in CMI six months after the operation, as the majority of patients failed to regain their pre-fracture mobility status (Table 7). According to our results, the majority of the patients were still lacking one or more levels of mobility six months after their treatment. The difference was found to be significant according to the Mann-Kendall test (*p* = 0.000). Only 8.3% of those who were assigned as 5/5 managed to maintain their previous mobility status, 37.5% lost one level of mobility, 33.3% two levels, and the remaining 4.2% and 16.7% had a mobility of 2/5 and 1/5, respectively, six months after the operative treatment. Details for the mobility status in other categories are available in Table 7.

## 4. Discussion

A hip fracture is a very significant injury to the human skeleton that dramatically affects the health-related quality of life of individuals after the aforementioned injury. In the literature, there is an increasing interest in the HRQoL of patients after surgical treatment of hip fractures, and a growing number of publications address this issue [11,17,18,19,20,24,25,26,27,28,29,30].

Our study aimed to assess the health-related quality of life and mortality in patients with intertrochanteric hip fracture treated with cephalomedullary nailing, as well as to explore possible risk and modifiable factors. The comparison is made between the values taken before the fracture and those measured six months after the surgical intervention for fracture fixation. To the best of our knowledge, this is the first study in Greece and South-East Europe that addresses HRQoL and mortality in patients with a fragility fracture. This study primarily includes women aged over 80, characterized by a low institutionalization index (5.4%), aligning with findings from other studies [18,20,26,29]. The mean total length of stay was 7.5 (1–23), which is considerably shorter than the 10 days reported in other studies [20] but longer compared to the 5.3 days found in others [17,18]. The use of the EQ-5D index as an assessment tool has limitations attributed to a bimodal distribution in the pre-operative EQ-5D index score [31]. Therefore, we also incorporated the EQ-5D VAS score and the SF-12 questionnaire.

### 4.1. HRQoL Measured by EQ-5D

Our findings affirm the negative impact of pertrochanteric hip fractures on quality of life, being in alignment with several publications that have examined the postoperative period of one to 12 months [11,17,28]. The baseline ED-5D index in our population was 0.74 (0.48–0.87), which is close to the corresponding index reported in other studies [18,20,27,32]. Most studies demonstrate an initial decline in the EQ-5D index [11,17,18,29], with significant improvement observed at 12 months compared to measurements typically taken at 1 or 4 months [11,17]. Our results at 6 months post-op indicate that the decline in EQ-5D remains significant, consistent with findings reported by other authors [18,20]. Amarilla-Donoso et al. suggested that the EQ-5D decline reaches a plateau after this period, with no further improvement observed at the 12-month follow-up [17]. Likewise, the ED-5D VAS follows a similar pattern, decreasing from the baseline of 69.7 (46–89) to 42.2 (28–61) six months post-operation based on our data. However, Amphansap and Sujarekul reported an initial value of 85 that dropped to 68 in the six-month follow-up [32], both significantly higher than our results. However, the results in this study may differ, as various treatment options were applied based on the patient’s health status and the type of fracture. In the study by Parsons et al., the initial EQ-5D VAS was comparable to ours (67.6), but the result at six months post-operation was lower than ours, measuring 33.2 [26].

### 4.2. HRQoL Measured by SF-12

Our study arrived at the same conclusion regarding HRQoL, observing a baseline value of 34.2 (27.3–54.5) for the physical component of SF-12 and 54.49 (39.5–71.7) for the mental component. Both values significantly declined to 27.6 (17.1–49.7) and 38.6 (27.2–67.3), respectively. These results are in complete accordance with the published findings of Amarilla-Danoso et al. [18]. Moerman et al. reported a similar decline in both components of SF-12 at three months postoperative and observed a recovery of the values at the twelve-month follow-up [19]. In this study, the MCS levels reached baseline levels at 12 months postoperatively. However, in our study, both MCS and PCS were still significantly lower compared to baseline levels six months after the provided surgical treatment. This discrepancy with our results may be attributed to the fact that our study exclusively included pertrochanteric fractures treated with cephalomedullary fixation. Similar to our findings, a statistically significant drop in SF-12 levels was reported by Amarilla-Donoso et al. six months post-operatively [17]. This study indicated that neither the mental nor the physical component reached the initial values at either the six- or twelve-month postoperative follow-up.

### 4.3. Factors Correlated to EQ-5D and SF-12

Our univariate analysis revealed no correlation between EQ-5D and age, CCI, ASA score, and rehabilitation, whether conducted at home or in a rehabilitation center. These results contrast with the findings of Amarilla-Danoso et al., who reported a significant correlation between age, CCI, previous hip fracture, baseline living status, polymedication, and depression [18]. In the study by Deutschbein et al., depressive symptoms, anxiety, pre-fracture dependency in day life activities, and the transfer to a rehabilitation center were found to be related to EQ-5D [20]. In contrast to our results in this study, the transfer to a rehabilitation facility resulted in a significantly better outcome in terms of EQ-5D [20]. Both of these studies, however, included patients with either pertrochanteric or intracapsular fractures treated with fixation or arthroplasty, which may have influenced their results in comparison to ours. In their study, van de Ree et al. demonstrated that one year after hospital admission for hip fracture, EQ-5D is negatively related to the frailty of patients. This relationship remains still significant after adjusting for age, ASA score, dementia, death, pre-fracture residential status, and mobility [28]. In another study conducted in Thailand, EQ-5D was found to be associated with age, body mass index (BMI), and operative treatment (as opposed to conservative treatment). Specifically, this study supported the idea that lower age, normal or high BMI, and operative treatment are positively correlated with the QoL measured by EQ-5D twelve months post-fracture [32].

In relation to the SF-12 index, our data analysis revealed a significant correlation between ASA score grading and SF-12. However, none of the other parameters mentioned above (age, CCI, rehabilitation) exhibited a significant impact on HRQoL measured by SF-12. In contrast, Moerman et al. reported that ASA score classifications I and II, pre-fracture mobility levels, and osteosynthesis treatment (compared to arthroplasty) were associated with greater decline in the physical component of SF-12 but not in mental component at three months post-operatively [19]. Sprague et al. reported that ASA score class III was correlated with lower physical QoL versus class I [33]. In their systematic literature review, Peeters et al. reported that insufficient nutritional status, female gender, existing medical issues, reduced physical or psychological functioning, and prolonged hospitalization periods are negatively associated with HRQoL [25].

### 4.4. Mortality and Mobility

Our 30-day mortality rate was 9.3%, with an annual mortality rate of 32.4%, both comparable to rates reported in other published studies [34,35]. Ngobeni reported an in-hospital mortality of 14% and a yearly mortality of 32% [35], while Roche et al. reported rates of 9.6% and 33.0%, respectively [34]. The gender distribution in the population of our study was similar to that reported in these studies, with a prevalence of female gender. However, some studies have reported lower one-year mortality rates compared to ours [17,36]. Wang et al. found a one-year mortality rate of 13.9% in patients aged above 90 years with intertrochanteric fractures treated with internal fixation [36]. Similarly, Amarilla-Donoso et al. reported a one-year mortality rate of 8.5% [17]. It is worth noting that almost one-third of the patients in Amarilla-Donoso et al.’s study were admitted with intracapsular neck of femur fractures, which may have contributed to different outcomes of mortality compared to other studies. In their study published by Deutschbein et al., the six-month mortality was 12%, with no substantial difference between males and females [20]. Our results may be influenced by the specific inclusion criteria, as we focused solely on pertrochanteric fractures treated with a cephalomedullary nail. Additionally, it is worth noting that our center, serving as a regional reference center, frequently manages a substantial caseload of severe and complicated fractures, as well as frail patients. The 30-days mortality rate in our study during the pandemic was significantly higher than in the pre-pandemic era. These results highlight a significant issue in the operation of the national health system during this period. Although the mean time elapsed from admission to surgical treatment was lower during the pandemic, the shortage of nursing staff and the system’s singular focus on one disease (COVID-19) may have resulted in a significant increase in 30-day mortality. Similar findings were reported in 2022 by Boukebous et al., where the 30-day survival was 97% in 2019 (compared to our rate of 95%) and 86% in 2020 (compared to our rate of 88%) [37]. Stitkitti et al. announced recently that the 1-year mortality rate after a fragility hip fracture was significantly higher during the pandemic outbreak, and this increase was not related to COVID-19 infection [38]. Vochteloo et al. conducted a study involving 390 patients over 65 years old who sustained a hip fracture. The percentage of those who managed to regain their pre-fracture mobility one year postoperatively was nearly 50% [39]. These results are considerably better compared to our findings in terms of postoperative mobility.

### 4.5. Factors Correlated to Mortality

In our study, ASA score was significantly correlated with 30-day mortality and anemia; age above 85 was found to be associated with 1-year mortality. Wang et al. found a significant correlation between 1-year mortality and time from injury to operation, respiratory failure, and anemia. However, in this study, ASA, age, and gender did not appear to have a significant impact on one year’s mortality [36]. These results are partially consistent with our findings. Greve et al. reported no correlation between waiting time to surgery and mortality [40], a finding similar to ours. Similar findings were reported by Ngobeni, who concluded that there was no disparity in mortality rates based on the timing of surgery, whether the patient underwent the procedure within less than 24 h or beyond 72 h, as long as the operations were conducted within a week [35]. However, Leer-Salvesen et al. have stated that a delay in operation plays a significant role in one-year mortality if it exceeds 48 h [41]. Low hemoglobin level on admission was found to significantly increase the risk of one-year mortality by 2.466 (*p* < 0.05), according to Greenhalgh et al. [42]. According to our data, the transfused group differed significantly from the non-transfused group in terms of age and hemoglobin levels (on admission and on discharge).

### 4.6. Strengths and Limitations

The strength of our study is the size of our sample, which is the largest in our country. This study is the first of its kind in South-East Europe. The response rate was 74%, which is considered adequate for this type of study and the duration of the follow-up. Our study has several limitations. One major limitation of this study is the inability to gather prospective information about the pre-fracture condition, which leads to the assumption of potential memory bias and a potential underestimation of the results. However, Howell et al. reported that patients could reliably recall their pre-operative functional status three months after a hip arthroplasty [43]. The incidence rate was calculated based on the number of patients operated on in our hospital, not the number admitted. Another major limitation of our study is the relatively low number of enrolled patients, as well as the fact that this is a single-center study. The additional limitation, in which patients in this study were exclusively interviewed via telephone rather than through face-to-face appointments, could be characterized as a further drawback. Additionally, this study does not explore the correlation between the type of fracture classification and its impact on quality or mortality. A radiographic assessment of fracture consolidation was not included, and the time needed for the union was not evaluated. Furthermore, patients with pre-fracture mobility limitations, such as Parkinson’s disease or stroke, were not excluded from the study population. Another limitation is the use of non-randomized sampling. However, in our study, all patients who met the inclusion criteria during the study period were included, leading to an approximate method. It is important to note that this approach does not guarantee the representativeness of the entire population. Another limitation lies in the possibility that alterations observed in Quality of Life (QoL) may not solely result from hip fracture but could be influenced by other unidentified factors occurring during the postoperative period. Finally, in our study, there is no comparison in terms of HRQoL, mortality, length of hospital stay, and transfusion rate between patients treated with closed reduction versus open reduction, as all of our patients were treated with short nails and the reduction was achieved in a closed or minimally invasive open manner. In more severe, subtrochanteric fractures, it seems to be beneficial to reduce the fracture in a closed manner instead of opting for an open reduction [44,45].

## 5. Conclusions

Health-related quality of life is significantly affected after a pertrochanteric hip fracture, remaining much lower even six months postoperatively compared to the pre-operative levels, according to SF-12 and EQ-5D. There appears to be a correlation between the ASA score and the decrease in quality when measured by SF-12. Annual mortality of these fractures remains high, and the pandemic has significantly increased the one-month mortality. The ASA score may serve as a predictor for the one-month mortality, as it exhibits a significant correlation with it. No significant difference was observed in the time elapsed from injury to operation between the mortality and survival groups. Further studies and assessments are needed to formulate a policy that will effectively manage and prevent excess mortality associated with this common type of injury among the elderly population.

## Figures and Tables

**Table 1:** (**a**) Clinical and sociodemographic characteristics (gender). (**b**) Clinical and sociodemographic characteristics (pandemic).

**(a)**				
**Patients Characteristics**	**All Patients**	**Female**	**Male**	***p***
Number (%)	259 (100)	212 (81.9)	47 (18.1)	
Age, mean (range)	83.6 (60–99)	84.0 (60–99)	81.7 (62–95)	0.626
ASA score, mean (range)	3 (2–4)	3.0 (2–4)	3.11 (2–4)	0.961
CCI, mean (SD)	5.7 (2–13)	5.7 (2–13)	6.0 (3–13)	0.245
Smoking (%)	42 (16.2)	24 (11.3)	18 (38.3)	<0.001
Non smoking	217 (83.8)	188 (88.7)	29 (61.7)	<0.001
Living in home pre Fx (%)	245 (94.6)	199 (93.9)	46 (97.9)	0.204
Living in facilities (%)	14 (5.4)	13 (6.1)	1 (2.1)	0.835
Osteoprotective Treatment pre-Fx (%)	10 (3.9)	10 (4.7)	0 (0.0)	-
Days to operation, mean (range)	2.7 (0–11)	2.6 (0–9)	3.1 (0–11)	0.047
Mean hospitalization time, Days (range)	7.5 (1–23)	7.4 (1–23)	8.3 (2–17)	0.538
**(b)**				
**Patients Characteristics**	**All Patients**	**Pre-COVID-19 Era**	**Post-COVID-19 Era**	***p***
Number (%)	259 (100)	101 (39%)	158 (61%)	
Age, mean (range)	83.6 (60–99)	83.9 (63–98)	83.3 (60–99)	0.790
ASA score, mean (range)	3 (2–4)	2.9 (2–4)	3.1 (2–4)	0.989
CCI, mean (range)	5.7 (2–13)	5.8 (3–13)	5.7 (2–13)	0.487
Smoking (%)	42 (16.2)	12 (12.0)	30 (19.0)	0.361
Non smoking	217 (83.8)	89 (88.0)	128 (81.0)	0.02
Living in home pre Fx (%)	245 (94.6)	98 (97.0)	147 (93.0)	0.159
Living in facilities (%)	14 (5.4)	3 (3.0)	11 (7.0)	0.604
Osteoprotective Treatment pre-Fx (%)	10 (3.9)	3 (3.0)	7 (4.4)	0.928
Days to operation, mean (range)	2.7 (0–11)	3.3 (0–11)	2.3 (0–9)	0.284
Mean hospitalization time, Days (range)	7.5 (1–23)	8.5 (4–17)	7.4 (1–23)	0.049
Rehabilitation Centre post op (%)	179 (69.1)	75 (74.3)	104 (65.8)	0.01
No rehab-centre post-op (%)	80 (30.9)	26 (25.7)	54 (34.2)	0.11

**Table 2:** (**a**) HRQoL pre-fracture and 6 months post-op. (**b**) HRQoL is in correlation with age, ASA, CCI, and post-op rehabilitation.

**(a)**				
	**Pre-Fracture**	**6 Months** **Post-Op**	***p***	**Effect Size**
EQ-5D index	0.74	0.39	<0.001	1.11
EQ-5D VAS	69.7	42.4	<0.001	2.46
SF-12 PCS	34.2	27.6	<0.001	1.21
SF-12 MCS	54.49	38.6	<0.001	1.02
**(b)**				
			**EQ-5D**	**SF-12**
Age	Spearman r		0.077	0.156
	*p*		0.438	0.116
ASA score	Spearman r		0.134	0.232
	*p*		0.178	0.018
CCI *	Spearman r		0.080	−0.018
	*p*		0.419	0.857
Own home	U		1009.5	1091.0
	*p*		0.543	0.977
Rehabilitation Center	U		864.0	944.5
	*p*		0.224	0.540

* Charlson Comorbidity Index.

**Table 3:** Mortality rates.

	All Patients	Female	Male	*p*	Pre-Pandemic	Pandemic	*p*
30-days mortality (%)	24 (9.3)	16 (7.5)	8 (17.0)	0.087	5 (5.0)	19 (12.0)	0.003
1-year mortality (%)	84 (32.4)	71 (33.5)	13 (27.7)	0.21	28 (27.7)	56 (35.4)	0.196

**Table 4:** Parameters correlated to mortality.

**30 Days Mortality**		
	**x^2^**	***p***
ASA	8.546	0.014
CCI	7.590	0.669
Gender	2.932	0.087
Transfusion	1.289	0.256
**1 Year Mortality**		
	**x^2^**	***p***
ASA	4.305	0.116
CCI	9.986	0.442
Gender	1.573	0.21
Transfusion	0.939	0.319

**Table 5:** Comparison of Possible Factors Associated with Mortality in 1 Year Follow-Up Between Two Groups.

Characteristics	Mortality Group (n = 84)	Survival Group (n = 175)	*p*	Effect Size
Age, years (range)	86.7 (66–99)	82.0 (60–98)	<0.001	0.62
Hemoglobin				
In Admission (g/dL)	11.5 (7–15.2)	12.0 (7.5–16.1)	<0.001	0.28
On Discharge (g/dL)	10.2 (7.5–12.4)	10.0 (8–13.5)	<0.001	0.1
Delay to operate (in days)	2.8 (0–8)	2.6 (0–11)	=0.091	0.09

**Table 6:** Comparison of Possible Factors Associated with Transfusion Between Two Groups.

Characteristics	Non Transfused (n = 87)	Transfused (n = 172)	*p*	Effect Size
Age, years (range)	81.3 (62–97)	84.7 (60–99)	<0.001	0.44
Hemoglobin				
Admision (g/gL)	12.9 (7.9–16.1)	11.4 (7–15.2)	<0.001	0.9
Discharge (g/dL)	10.7 (8.4–13.5)	9.8 (7.5–12.5)	<0.001	0.45
Delay to operate in days	2.6 (0–8)	2.7 (0–11)	0.093	0.045

**Table 7:** Patients’ mobility pre-FX and post-op (in percentages).

		Post-Op Mobility				
		1/5	2/5	3/5	4/5	5/5
Pre-FX mobility	1/5	100.0%	0.0%	0.0%	0.0%	0.0%
	2/5	40.0%	40.0%	0.0%	20.0%	0.0%
	3/5	14.8%	44.4%	37.0%	3.7%	0.0%
	4/5	9.5%	19.0%	57.1%	14.3%	0.0%
	5/5	16.7%	4.2%	33.3%	37.5%	8.3%

## Data Availability

Data is unavailable due to privacy and ethical restrictions.
